# Partial Volume Correction Increases the Sensitivity of 18F-Florbetapir-Positron Emission Tomography for the Detection of Early Stage Amyloidosis

**DOI:** 10.3389/fnagi.2021.748198

**Published:** 2021-12-22

**Authors:** Stefan J. Teipel, Martin Dyrba, Andrea Vergallo, Simone Lista, Marie Odile Habert, Marie-Claude Potier, Foudil Lamari, Bruno Dubois, Harald Hampel, Michel J. Grothe

**Affiliations:** ^1^German Center for Neurodegenerative Diseases (DZNE), Rostock, Germany; ^2^Department of Psychosomatic Medicine, University Medicine Rostock, Rostock, Germany; ^3^Sorbonne University, GRC n° 21, Alzheimer Precision Medicine (APM), AP-HP, Pitié-Salpêtrière Hospital, Boulevard de l’Hôpital, Paris, France; ^4^Brain & Spine Institute (ICM), INSERM U 1127, CNRS UMR 7225, Boulevard de l’Hôpital, Paris, France; ^5^Department of Neurology, Institute of Memory and Alzheimer’s Disease (IM2A), Pitié-Salpêtrière Hospital, AP-HP, Boulevard de l’Hôpital, Paris, France; ^6^Laboratoire d’Imagerie Biomédicale, CNRS, INSERM, LIB, Sorbonne University, Paris, France; ^7^Department of Nuclear Medicine, Pitié-Salpêtrière Hospital, AP-HP, Paris, France; ^8^Centre d’Acquisition et Traitement des Images (CATI platform), Paris, France; ^9^ICM Institut du Cerveau et de la Moelle Épinière, CNRS UMR 7225, INSERM U1127, UPMC, Hôpital de la Pitié-Salpêtrière, 47 Bd de l’Hôpital, Paris, France; ^10^UF Biochimie des Maladies Neurométaboliques, Service de Biochimie Métabolique, Hôpital Pitié-Salpêtrière, Paris, France; ^11^Unidad de Trastornos del Movimiento, Servicio de Neurología y Neurofisiología Clínica, Instituto de Biomedicina de Sevilla, Hospital Universitario Virgen del Rocío/CSIC/Universidad de Sevilla, Seville, Spain

**Keywords:** amyloid PET, cerebral amyloidosis, partial volume effect, cerebrospinal fluid, Alzheimer’s disease, early detection

## Abstract

**Purpose:** To test whether correcting for unspecific signal from the cerebral white matter increases the sensitivity of amyloid-PET for early stages of cerebral amyloidosis.

**Methods:** We analyzed 18F-Florbetapir-PET and cerebrospinal fluid (CSF) Aβ42 data from 600 older individuals enrolled in the Alzheimer’s Disease Neuroimaging Initiative (ADNI), including people with normal cognition, mild cognitive impairment (MCI), and Alzheimer’s disease (AD) dementia. We determined whether three compartmental partial volume correction (PVC-3), explicitly modeling signal spill-in from white matter, significantly improved the association of CSF Aβ42 levels with global 18F-Florbetapir-PET values compared with standard processing without PVC (non-PVC) and a widely used two-compartmental PVC method (PVC-2). In additional voxel-wise analyses, we determined the sensitivity of PVC-3 compared with non-PVC and PVC-2 for detecting early regional amyloid build-up as modeled by decreasing CSF Aβ42 levels. For replication, we included an independent sample of 43 older individuals with subjective memory complaints from the INveStIGation of AlzHeimer’s PredicTors cohort (INSIGHT-preAD study).

**Results:** In the ADNI sample, PVC-3 18F-Florbetapir-PET values normalized to whole cerebellum signal showed significantly stronger associations with CSF Aβ42 levels than non-PVC or PVC-2, particularly in the lower range of amyloid levels. These effects were replicated in the INSIGHT-preAD sample. PVC-3 18F-Florbetapir-PET data detected regional amyloid build-up already at higher (less abnormal) CSF Aβ42 levels than non-PVC or PVC-2 data.

**Conclusion:** A PVC approach that explicitly models unspecific white matter binding improves the sensitivity of amyloid-PET for identifying the earliest stages of cerebral amyloid pathology which has implications for future primary prevention trials.

## Introduction

Cerebrospinal fluid (CSF) concentrations of the 42-amino acid-long amyloid-β peptide (Aβ42) (Blennow et al., 2015) and amyloid-sensitive positron emission tomography (PET) tracers (Zhang et al., 2014; Martinez et al., 2017) serve as disease-defining markers of Alzheimer’s disease (AD) in recently revised diagnostic research criteria (Jack et al., 2018). CSF Aβ42 levels have been found to be strongly associated with global signal from amyloid-sensitive PET using 11C-PIB (Leuzy et al., 2016), 18F-Florbetaben (Spallazzi et al., 2019), 18F-Flutemetamol (Janelidze et al., 2016), and 18F-Florbetapir (Toledo et al., 2015) tracers. However, the association of CSF Aβ42 levels with global amyloid-PET signal has been described as non-linear, being strong in the middle range of values, but weak or absent in the lower range of amyloid levels (Toledo et al., 2015; Leuzy et al., 2016; Palmqvist et al., 2016; Kalheim et al., 2018). Based on these observations it has been proposed that amyloid-PET may have a lower sensitivity for early stages of cerebral amyloidosis than CFS Aβ42 (Palmqvist et al., 2016).

However, the sensitivity of amyloid-PET may critically depend on the way the PET scans are being analyzed. A particular concern for the quantification of amyloid-PET signal is the so called partial volume effect (PVE). The PVE results from two related mechanisms reflecting the poor spatial resolution of PET scans relative to the thickness of the cerebral gray matter (Hoffman et al., 1979). First, the limited spatial resolution leads to displacement of activity counts between neighboring regions, resulting in spill-out of gray matter signal into surrounding low intensity areas (particularly CSF) as well as spill-in of unspecific binding signal from the white matter. In addition, due to sampling effects the relatively large voxel size leads to a mixture of tissue classes (e.g., gray matter, white matter and CSF) within a single voxel (Erlandsson et al., 2012). Due to the characteristically high non-specific white matter binding in amyloid-sensitive PET imaging the net PVE will depend on the actual cortical amyloid load: in early stage amyloidosis, when the actual cortical tracer concentration is relatively small, the measured signal will be highly influenced by spill-in of non-specific white matter signal, whereas in advanced stage amyloidosis, when actual cortical tracer concentration approaches or surpasses the non-specific white matter signal, cortical signal loss due to spill-out becomes more relevant (Matsubara et al., 2016; Gonzalez-Escamilla et al., 2017; Lopez-Gonzalez et al., 2019).

Partial volume effect correction (PVC) methods are designed to decrease the contribution of unspecific signal, and would thus be expected to increase the sensitivity of amyloid-PET for lower levels of amyloid accumulation, particularly when explicitly accounting for spill-in of white matter signal. Indeed, PVC using a 3-compartment approach (Müller-Gärtner et al., 1992) was found to increase the accuracy of 11C-PIB (Mikhno et al., 2008), 18F-Florbetaben-PET (Rullmann et al., 2016), and 18F-Florbetapir-PET (Gonzalez-Escamilla et al., 2017) to discriminate between AD patients and healthy controls. In addition, PVC increased the sensitivity of 18F-Florbetaben-PET for the detection of postmortem Aβ plaque load in 31 cases with antemortem PET and postmortem autopsy (Rullmann et al., 2016).

Here, we explored the effect of different image processing strategies on the association of 18F-Florbetapir-PET signal with CSF Aβ42 levels as reference measure of overall amyloid burden, using combined PET and CSF data of 600 older individuals enrolled in the Alzheimer’s Disease Neuroimaging Initiative (ADNI), including individuals with normal cognition, mild cognitive impairment (MCI), and AD dementia. To test the replicability of our findings in an independent cohort, we used data from 43 cognitively normal individuals with subjective memory complaints (SMC) from the monocentric INveStIGation of AlzHeimer’s PredicTors in subjective memory complainers (INSIGHT-preAD) cohort (Dubois et al., 2018). Our primary objective was to investigate whether 3-compartment PVC (Müller-Gärtner et al., 1992) improves the association between 18F-Florbetapir-PET signal and CSF Aβ42 levels, with a particular focus on the early stages of amyloid build-up. We also assessed these associations when using a widely-employed 2-compartment PVC approach (Meltzer et al., 1996) that does not account for possible white matter spill-in, and further examined the effect of using a white matter reference region to calculate PET standard uptake value ratio (SUVR) instead of the whole cerebellum reference standard (Lopez-Gonzalez et al., 2019). In additional voxel-based analyses, we determined the relative sensitivities of the different processing strategies to detect initial regional 18F-Florbetapir-PET signal elevations at early signs of cerebral amyloidosis as modeled by decreasing CSF Aβ42 levels. More sensitive detection of early stage amyloid build-up using PET would help identifying people at increased risk for AD dementia in very early stages, a cohort that is highly relevant for testing the effect of preventive treatments.

## Materials and Methods

### Data Source

Data used in the preparation of this article were obtained from the ADNI database.^1^ The ADNI was launched in 2003 by the National Institute on Aging, the National Institute of Biomedical Imaging and Bioengineering, the Food and Drug Administration, private pharmaceutical companies and non-profit organizations, with the primary goal of testing whether neuroimaging, neuropsychological, and other biologic measurements can be used as reliable *in vivo* markers of AD pathogenesis. A fuller description of ADNI and up-to-date information is available at www.adni-info.org.

To test if key findings could be replicated in an independent sample, we used data from the INSIGHT-preAD study (Dubois et al., 2018). This study is a monocentric university based cohort of participants enrolled at the Institute of Memory and Alzheimer’s Disease (IM2A) at the Pitié-Salpêtrière University Hospital in Paris, France. The main objective is to investigate the earliest preclinical stages of AD and its development including influencing factors and markers of progression.

### Study Participants

From the ADNI cohort, we retrieved 600 cases with available 18F-Florbetapir-PET data as well as CSF Aβ42 levels in close temporal proximity to the PET acquisition. Mean temporal distance between 18F-Florbetapir-PET acquisition and lumbar puncture was 13 days (SD 34 days). These cases included data of 152 cognitively normal older individuals, 241 cases with early MCI, 129 subjects with late MCI and 78 subjects with AD dementia. Detailed inclusion criteria for the diagnostic categories are available at the ADNI web site.^2^

The INSIGHT-preAD study includes 318 cognitively normal Caucasian individuals, recruited from the community in the wider Paris area, aged 70–85 at baseline, with SMC and defined brain amyloid status (Dubois et al., 2018). Forty-three INSIGHT-preAD cases had CSF Aβ42 levels available in close temporal proximity to the 18F-Florbetapir-PET scan. These cases represented the replication cohort. Details on participants’ demographics for both samples are shown in Table 1.

**TABLE 1:** Participants’ demographics.

ADNI cohort					

	f/m^a^	Age (SD) (years)^b^	MMSE (SD)^c^	Education years (SD)^d^	CSF Aβ42^§^ (SD) (pg/ml)^e^
CN	78/74	73.6 (6.5)	29.1 (1.1)	16.6 (2.5)	1395.2 (665.5)
EMCI	132/109	71.4 (7.4)	28.4 (1.6)	15.9 (2.7)	1206.9 (593.6)
LMCI	69/60	72.2 (7.9)	27.5 (1.9)	16.6 (2.7)	927.7 (499.5)
AD	45/33	75.7 (8.6)	23.0 (2.0)	15.5 (2.8)	696.7 (410.8)

**INSIGHT-preAD cohort**					

	**f/m**	**Age (SD) [years]**	**MMSE (SD)**	**max. primary/min. secondary education**	**CSF Aβ42^§^ (SD) [pg/ml]**

SMC	21/22	74.9 (3.3)	28.8 (0.9)	7/36	885.8 (362.7)

*^a^Not significantly different across groups, Chi^2^ = 0.94, 3 df, p = 0.82.*

*^b^Significantly different across groups, ANOVA, F(3, 598) = 7.6, p < 0.0001.*

*^c^Significantly different across groups, Kruskal-Wallis ANOVA, p < 0.0001.*

*^d^Significantly different across groups, ANOVA, F(3, 598) = 5.23, p < 0.001.*

*^e^Significantly different across groups, ANOVA, F(3, 598) = 32.4, p < 0.0001.*

*^§^CSF Aβ42 values from electrochemiluminescence immunoassays on an automated Elecsys cobas e 601 instrument.*

*^$^CSF Aβ42 values from a double antibody sandwich ELISA method (Innotest-Fujirebio^®^, Courtaboeuf, France).*

*CN, healthy controls; EMCI, early MCI cases; LMCI, late MCI cases; AD, AD dementia cases; SMC, subjective memory complaints.*

*For definition of diagnostic categories see “Study Participants” section of the main text.*

As described in the ethics declaration section below, written informed consent was obtained from all participants and/or authorized representatives before any protocol-specific procedures were carried out.

### Cognitive Tests

ADNI and INSIGHT-preAD cases underwent comprehensive neuropsychological examinations. In the present study, the Mini Mental State Examination (MMSE) (Folstein et al., 1975) was used to assess global cognition across both samples.

### Cerebrospinal Fluid Measurements

ADNI CSF values in the current study were derived from electrochemiluminescence immunoassays for Aβ (1-42) on an automated Elecsys cobas e 601 instrument. The upper technical limit for this assay is 1,700 pg/ml and the values that are higher than 1,700 pg/ml are based on an extrapolation of the calibration curve. Therefore, analyses were repeated with CSF Aβ42 values from the previously used multiplex xMAP Luminex platform (Luminex Corp, Austin, TX, United States) with INNOBIA AlzBio3 kit (Innogenetics, Ghent, Belgium) (Shaw et al., 2009).

INSIGHT-preAD CSF Aβ42 values were determined using a double antibody sandwich ELISA method (Innotest-Fujirebio^®^, Courtaboeuf, France).

### Imaging Data Acquisition

Detailed acquisition and standardized pre-processing steps of ADNI imaging data are available at the ADNI website (see text footnote 2). 18F-Florbetapir-PET data was collected during a 50- to 70-min interval following a 370 MBq bolus injection of 18F-Florbetapir. To account for the multicentric acquisition of the data across different scanners and sites, all PET scans undergo standardized pre-processing steps within ADNI.

PET data acquisition in the INSIGHT-preAD cohort was performed according to a method previously described (Habert et al., 2017). All amyloid-PET scans were acquired in a single session on a Philips Gemini GXL CT-PET scanner 50 (±5) minutes after the injection of approximately 370 MBq (333-407 MBq) of 18F-Florbetapir (AVID radiopharmaceuticals).

For anatomical reference and pre-processing of the PET scans we used the corresponding structural MRI scan that was closest in time to the 18F-Florbetapir-PET scan. In ADNI, MRI scans were acquired on multiple 3T MRI scanners using scanner-specific T1-weighted sagittal 3D MPRAGE sequences. Similar to the PET data, MRI scans undergo standardized preprocessing steps aimed at increasing data uniformity across the multicenter scanner platforms (see text footnote 2 for detailed information on multicentric MRI acquisition and preprocessing in ADNI). In INSIGHT-preAD, MRI scans were acquired on a Siemens Verio 3T scanner at Pitié-Salpêtrière Hospital, Paris. A T1-weighted image was acquired using a fast three dimensional gradient echo pulse sequence using a magnetization preparation pulse (Turbo FLASH) (Habert et al., 2017).

### Positron Emission Tomography Data Pre-processing and Analysis

Images were preprocessed using Statistical Parametric Mapping software version 8 (SPM8) (The Wellcome Trust Centre for Neuroimaging, Institute of Neurology, University College London) implemented in Matlab 2013. The pre-processing pipeline followed a routine described previously (Grothe et al., 2017). First, each subject’s averaged PET frames were co-registered to their corresponding T1-weighted MRI scan. PVE were corrected in native space using two approaches, a 3-compartmental voxel-based post-reconstruction method as described by Müller-Gärtner et al. (1992) (henceforth termed PVC-3) and a simpler 2-compartmental approach as proposed by Meltzer et al. (1996) (henceforth termed PVC-2). Both PVC approaches are implemented in the SPM-based PETPVE12 toolbox (Gonzalez-Escamilla et al., 2017). The uncorrected and corrected PET images were spatially normalized to an aging/AD-specific reference template using the deformation parameters derived from the normalization of their corresponding MRI.

The composition of the cortical SUV mask has been described in Teipel et al. (2014). In brief, this composite mask consisted of frontal, parietal, and temporal ROIs known to be vulnerable to amyloid accumulation (Thal et al., 2002). Anatomical masks for these ROIs were derived from the Harvard-Oxford structural atlas (distributed with the software package, FSL; Desikan et al., 2006) and high-dimensionally warped into the reference space of this study based on a DARTEL registration of the MNI152 template (the template space of the Harvard-Oxford atlas) to the aging-AD reference template. SUVR values were calculated for the cortical composite region in uncorrected and PVC data by dividing the mean uptake values by the mean uptake value of a whole cerebellar reference region (in uncorrected data). In additional analyses, we used an alternative white matter reference region consisting of a binarized template of the segmented white matter tissue probability map of the reference template (>0.99 probability). In total, this resulted in six types of SUVR values arising from three different correction methods (no PVC, PVC-2, PVC-3) by two different reference regions (whole cerebellum, white matter). The most widely established standard approach corresponds to SUVRs from no PVC PET data using whole cerebellum as reference region (Klunk et al., 2015; Catafau et al., 2016; Navitsky et al., 2018). In the replication cohort from INSIGHT-preAD, only no PVC and PVC-3 were compared using whole cerebellum as a reference region.

For additional voxel-wise analyses, spatially normalized 18F-Florbetapir-PET scans (no PVC, PVC-2, PVC-3) were converted to voxel-wise SUVRs by scaling each voxel to the whole cerebellum (WC) reference signal (in uncorrected data). 18F-Florbetapir-PET SUVR_WC_ maps were masked to exclude non-gray matter voxels and smoothed using a Gaussian kernel of 10 mm^3^ × 10 mm^3^ × 10 mm^3^. Due to the low number of cases in the replication cohort, the complementary voxel-wise analyses were only conducted in the ADNI sample.

### Statistical Analysis

Associations between global 18F-Florbetapir-PET SUVR values and CSF Aβ42 levels were determined using two complementary approaches. First, we used spline regression to model non-linear associations between SUVR values and CSF Aβ42 levels. We decided *a priori* to use two knots fixed at the first and second tertile of the range of SUVR values. This decision was made based on the notion that we wanted to analyze three levels of amyloid: low, intermediate and high. In a sensitivity analysis focused on the cases with low amyloid levels, we iterated linear spline estimates for the first knot sliding in 100 steps from the 16.6% quantile to the first tertile of the SUVR values. Of note, for this analysis the overall number of knots of the spline regression was unimportant, as this analysis took only the first knot into account. To render spline estimates comparable across PVC approaches and employed reference regions, the SUVR values and CSF Aβ42 levels were scaled to be mean-centered and have a standard deviation of one before spline regression. Secondly, we determined Pearson’s product moment correlations between SUVR values and CSF Aβ42 levels across the entire sample as well as within tertiles of SUVR values. Correlation coefficients from different PVC methods and reference regions were compared with the correlation coefficients from the standard processing (no PVC, whole cerebellum reference) using Steiger’s Z-test. Again, for sensitivity analysis, we determined the distribution of correlation coefficients when cases were selected sliding in 100 steps from the 16.6% quantile to the first tertile of SUVR values.

These analyses were performed with RStudio, version 1.1.463, a user interface of R Project for Statistical Computing Analyses. Spline regression was conducted using the commands “lspline” and “qlspline” from library “lspline,” and Steiger’s Z-test was conducted using the command “r.test” from library “psych.” The libraries used are available at http://cran.r-project.org/web/packages.

To detect regional 18F-Florbetapir-PET SUVR increases in early amyloidosis, we used an approach inspired by Villeneuve et al. (2015). The authors used a voxel-wise sliding window analysis to determine a more sensitive global signal threshold for PiB-PET positivity at which first regional effects become evident. Following this approach, we ranked all healthy controls by their CSF Aβ42 levels and selected the 15% quantile (*n* = 23) with the highest CSF Aβ42 levels as the reference group. We then compared a series of subsequent groupings of the remaining cases with this reference group as follows. After selecting the 23 cases that comprised the reference group, we took the 23 subjects with the next lower CSF Aβ42 levels and performed voxel-wise contrasts between this group of interest and the reference group. We then dropped the three cases with the highest CSF Aβ42 levels from the group of interest and added the three subjects with the next lowest values and again performed a voxel-wise comparison with the reference group. We continued this process, iteratively creating groups of interest of 23 subjects by dropping the three cases with the highest CSF Aβ42 levels and adding the three cases with the next lowest levels, such that each new group of interest differed from the previous group by only six subjects. In this way, groups of interest gradually moved down the scale of CSF Aβ42 levels, always using the same initial reference group of 23 cases for comparison. This procedure was repeated 36 times until the three healthy controls with the lowest CSF Aβ42 levels were included. For each comparison, voxel-wise two-sample t-tests were performed using SPM12 software with the spatially normalized 18F-Florbetapir-PET SUVR maps from the different PVE correction methods. All voxel-wise analyses were corrected for multiple comparisons using a family-wise error (FWE) at *p* < 0.05 and cluster size *k* = 150. With this approach we addressed two questions: First, does the PVC method affect the sensitivity of 18F-Florbetapir-PET for early signs of cerebral amyloidosis as indicated by CSF Aβ42 levels? Second, where does regional amyloid signal occur first in people with low levels of amyloidosis?

Since the selection of the upper 15% quantile of CSF Aβ42 levels for the reference group was purely arbitrarily (with the goal to achieve a reasonable group size), in sensitivity analyses we used other cut-offs, specifically the 20% (*N* = 30; with a slide of 5 cases) and 10% quantiles (*N* = 15; with a slide of 2 cases), respectively. In addition, we applied this approach to all 600 cases, irrespective of diagnosis, with a 15% quantile cut-off (resulting in a group size of 90 cases) and a slide of 5 cases per window.

## Results

### Demographics

As shown in Table 1, within the ADNI sample groups differed significantly in age, education and, as expected, in MMSE scores and CSF Aβ42 levels, but not in sex distribution.

### Association of Global 18F-Florbetapir-Positron Emission Tomography Signal With Cerebrospinal Fluid Aβ42 Levels

**FIGURE 1:**
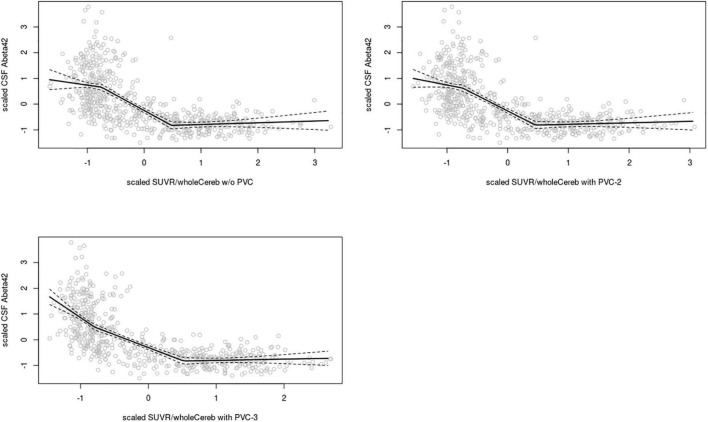
Linear spline estimates for CSF Aβ42 (Elecsys assay) vs. 18F-Florbetapir-PET SUVR in the ADNI sample, whole cerebellum reference. Spline regression with 95% confidence interval (dashed lines) of 18F-Florbetapir-PET SUVR_WC_ values on CSF Aβ42 levels using knots fixed at the first and the second tertiles of the range of 18F-Florbetapir-PET values. The 18F-Florbetapir-PET and CSF Aβ42 levels were scaled to a mean of 0 and a standard deviation of 1 to render models comparable across PVC methods and reference regions. Upper left: w/o (without) PVC, Upper right: PVC-2, Lower left: PVC-3.

**FIGURE 2:**
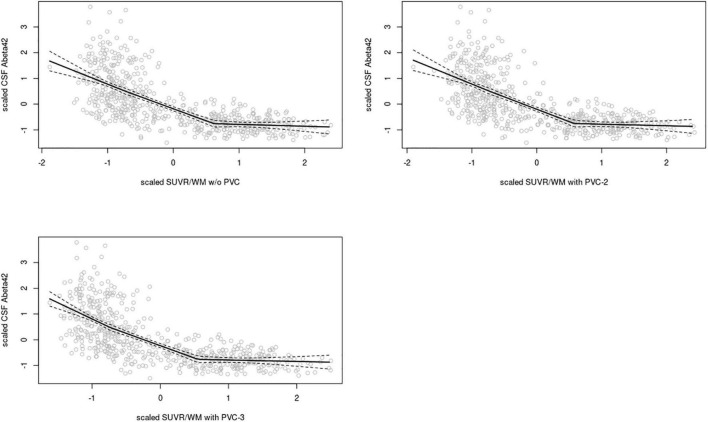
Linear spline estimates for CSF Aβ42 (Elecsys assay) vs. 18F-Florbetapir-PET SUVR in the ADNI sample, white matter reference. Spline regression with 95% confidence interval (dashed lines) of 18F-Florbetapir-PET SUVR_*WM*_ values on CSF Aβ42 levels using knots fixed at the first and the second tertiles of the range of 18F-Florbetapir-PET values. The 18F-Florbetapir-PET and CSF Aβ42 levels were scaled to a mean of 0 and a standard deviation of 1 to render models comparable across PVC methods and reference regions. Upper left: w/o (without) PVC, Upper right: PVC-2, Lower left: PVC-3.

**FIGURE 3:**
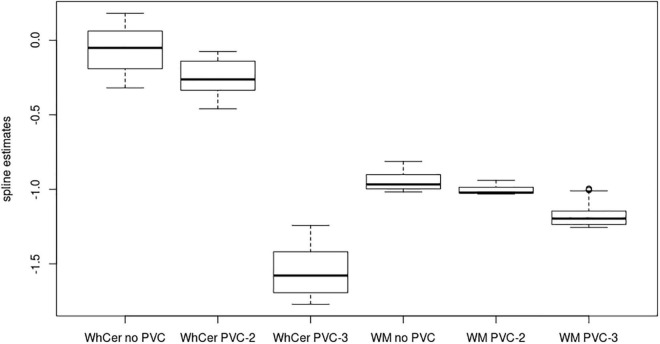
Linear spline coefficients for early-stage amyloidosis in the ADNI sample. Boxplots of linear spline coefficients from the regression of the 18F-Florbetapir-PET SUVR values on CSF Aβ42 levels with the range sliding between the 16% quantile and the first tertile of 18F-Florbetapir-PET values. WhCer, whole cerebellum reference region; WM, white matter reference region.

**FIGURE 4:**
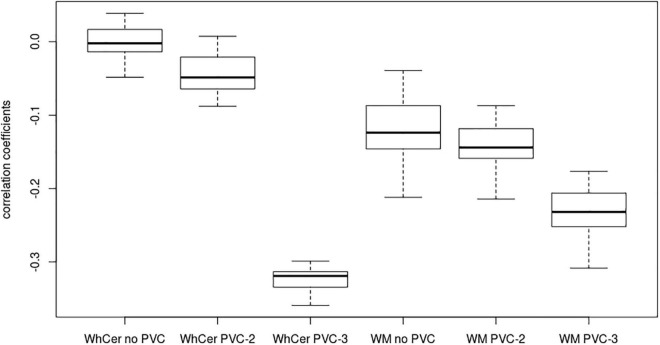
Correlation coefficients for early-stage amyloidosis in the ADNI sample. Boxplots of correlation coefficients between 18F-Florbetapir-PET SUVR values and CSF Aβ42 levels with the range sliding between the 16% quantile and the first tertile of 18F-Florbetapir-PET values. WhCer, whole cerebellum reference region; WM, white matter reference region.

In the **ADNI sample**, plots of linear spline regressions with fixed knots at the first and the second tertile of SUVR_WC_ values indicated a steeper slope for the first tertile when using PVC-3 compared to PVC-2 or no PVC (Figure 1); this effect was less evident for the white matter reference (Figure 2). To determine if this effect depended on the choice of the knot position, we allowed the values of the first knot to slide from the 16.6% quantile of the SUVR distribution to the first tertile. The boxplots in Figure 3 summarize the resulting distributions of linear spline estimates for this low range of SUVR values for the different PVC methods and reference regions. The lack of overlap of the boxes for PVC-3 with the other boxes indicates a significantly more negative association between CSF Aβ42 levels and low range 18F-Florbetapir-PET SUVR values when using PVC-3 as compared to PVC-2 and no PVC. The same effect can be seen in Figure 4 for the boxplots of the correlation coefficients, as the selected cases slided between the 16.6% and the 33.3% quantile of SUVR values. Table 2 lists the correlation coefficients for all tertiles of the SUVR distribution for each of the processing strategies. Across tertiles, statistical testing using Steiger’s Z test on Fisher’ z transformed correlation coefficients revealed significantly higher negative correlations when using PVC-3 SUVR_WC_ values as compared to the standard processing without PVC, and this effect was particularly pronounced in the lowest tertile (*r* = −0.34 for PVC-3 vs *r* = −0.01 for no PVC). By contrast, correlation coefficients for PVC-2 did not differ from the values obtained by the standard processing. For the white matter reference, correlations were significantly more negative than for the standard processing, except for the middle tertile, and correlation coefficients were comparable across PVC methods in this data (Table 2). The results for linear spline estimates and correlations were fully replicated using the CSF Aβ42 values derived from the Innogenetics ELISA assay (see sensitivity analyses in the Supplementary Material).

**TABLE 2:** Association between global 18F-Florbetapir-PET SUVR and CSF Aβ42 levels in the ADNI sample.

Reference region	Whole cerebellum			White matter		
		
Correction method	No PVC	PVC-2	PVC-3	No PVC	PVC-2	PVC-3
Total range	−0.62	−0.61	−0.67***	−0.68***	−0.68***	−0.69***
Low SUVR (1st tertile)	−0.01	−0.02	−0.34***	−0.21^$^	−0.21^$^	−0.31*
Middle SUVR (2nd tertile)	−0.44	−0.43	−0.58***	−0.53	−0.50	−0.54
High SUVR (3rd tertile)	0.07	0.08	−0.04*	−0.23**	−0.18*	−0.21**

^$^/*/**/*** = Correlation coefficient significantly different from correlation using standard SUVR method (no PVC, whole cerebellum reference), Steiger’s Z test, p < 0.05/0.01/0.001/0.0001.

In the **replication sample**, linear spline regression plots again indicated a steeper slope for the first tertile of SUVR_WC_ values when using PVC-3 compared to no PVC (Supplementary Figure 3). Correspondingly, the correlation between SUVR_WC_ values and CSF Aβ42 levels was significantly more negative for the PVC-3 than for the no PVC data across all cases [r(CSF/PVC-3) = −0.62, r(CSF/no PVC) = −0.48, t(43) = 2.5, *p* < 0.02]. Numerically, the correlation coefficient for PVC-3 was also markedly more negative than for no PVC data within the first tertile of data, but this effect did not reach statistical significance in this small subsample [*n* = 14; r(CSF/PVC-3) = −0.50, r(CSF/no PVC) = −0.13, *t*(14) = 1.1, *p* = 0.31].

### Regional Detection of Early Cerebrospinal Fluid Aβ42 Amyloidosis

**FIGURE 5:**
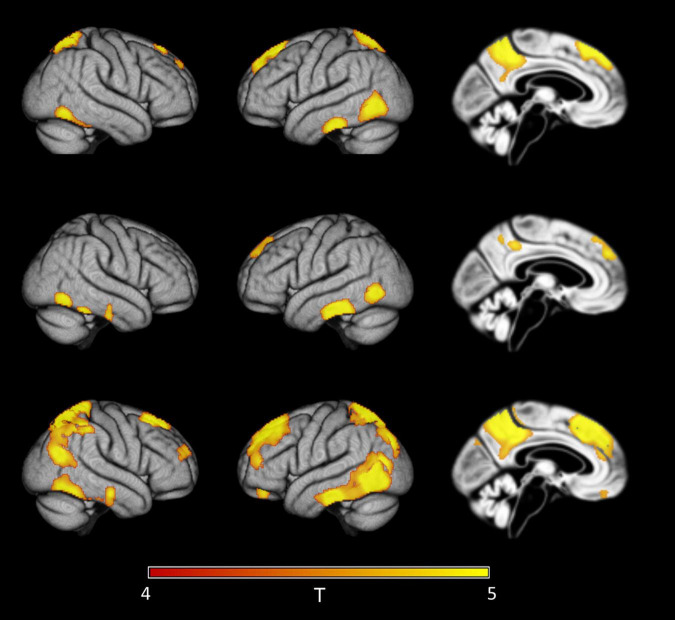
Detection of early regional amyloid accumulation in cognitively healthy older individuals. Effect of partial volume correction (PVC) method on first detectable signal increases in voxel-wise 18F-Florbetapir-PET SUVR_WC_ values as determined by the sliding-window analysis with decreasing CSF Aβ42 levels in cognitively healthy controls. Clusters of >150 voxels passing a family wise error corrected level of significance of *p* < 0.05 are plotted on rendered cortical surfaces and a midsagittal brain section. Effects are shown for the index sample (group mean CSF Aβ level of 840 pg/ml) where first regional signal increases compared to the reference sample (group mean CSF Aβ level of 2,512 pg/ml) were detectable in standard uncorrected 18F-Florbetapir-PET data (‘no PVC’). Across PVC methods regional effects appear in similar locations but are much more widespread in PVC-3 data (last row) compared to no PVC (first row) or PVC-2 data (second row).

When considering only cognitively healthy people, first regional effects in standard uncorrected 18F-Florbetapir-PET SUVR_WC_ maps were detected at a group mean CSF Aβ42 level of 840 pg/ml, and localized to the inferior temporal and temporo-occipital cortex, precuneus, superior parietal lobule, and medial prefrontal cortical areas (Figure 5). In PVC-3 corrected 18F-Florbetapir-PET SUVR_WC_ maps, first significant signal increases appeared in largely the same cortical regions but were already detectable at a group mean CSF Aβ42 level of 928 pg/ml. In PVC-2 corrected 18F-Florbetapir-PET SUVR_WC_ maps, first regional effects became detectable at the same group mean CSF Aβ42 level (840 pg/ml) as for the standard processing. These results were replicated using the 10th and the 20th percentiles for creating the reference samples (see Sensitivity Analyses in the Supplementary Material).

**FIGURE 6:**
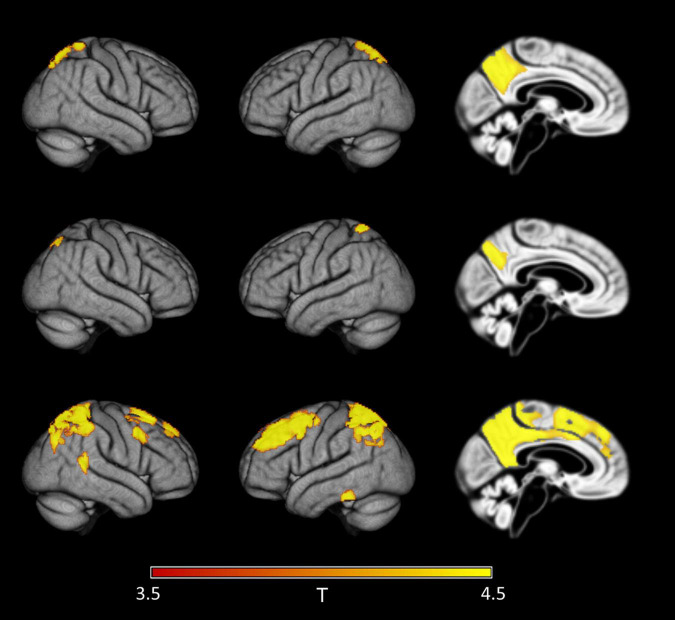
Detection of early regional amyloid accumulation across all cases of the ADNI sample. Effect of partial volume correction (PVC) method on first detectable signal increases in voxel-wise 18F-Florbetapir-PET SUVR_WC_ values as determined by the sliding-window analysis with decreasing CSF Aβ42 levels across all cases in the ADNI sample. Clusters of >150 voxels passing a family wise error corrected level of significance of *p* < 0.05 are plotted on rendered cortical surfaces and a midsagittal brain section. Effects are shown for the index sample (group mean CSF Aβ level of 1,287 pg/ml) where first regional signal increases compared to the reference sample (group mean CSF Aβ level of 2,280 pg/ml) were detectable in standard uncorrected 18F-Florbetapir-PET data (‘no PVC’). Across PVC methods regional effects appear in similar locations but are much more widespread in PVC-3 data (last row) compared to uncorrected (first row) or PVC-2 (second row) data.

When considering all ADNI cases irrespective of diagnosis, regional distribution of first significant effects were similar to the analysis including only healthy controls. Again, sensitivity to detect these effects was higher for PVC-3 (group mean CSF Aβ42 level of 1,376 pg/ml) than for no PVC and PVC-2 (group mean CSF Aβ42 level of 1,287 pg/ml for both) (Figure 6).

## Discussion

We determined the effect of different PVC methods and reference regions on the correspondence of the 18F-Florbetapir-PET signal with CSF Aβ42 levels and on the sensitivity of 18F-Florbetapir-PET to detect early build-up of regional amyloid. Amyloid-sensitive PET tracers, including 18F-Florbetapir, show high unspecific binding in cerebral white matter (Schmidt et al., 2015). This leads to a proportionally higher confound of cortical amyloid signal at low levels of specific uptake so that accounting for this effect was expected to particularly increase the sensitivity of 18F-Florbetapir-PET for early amyloid build-up. Consistent with this assumption, we found that 3-compartment PVC (Müller-Gärtner et al., 1992) of 18F-Florbetapir-PET SUVR_WC_ values, taking spill-in of white matter signal into account, but not 2-compartmental PVC (Meltzer et al., 1996), significantly increased the association of 18F-Florbetapir-PET signal with CSF Aβ42 levels, and this effect was most pronounced within the lower range of global amyloid levels. The effect was also stable across both the ADNI sample and the INSIGHT-preAD replication sample as well as across different CSF Aβ42 assays. In complementary voxel-wise analyses, PVC-3 also increased the sensitivity of 18F-Florbetapir-PET to detect first signs of regional amyloid build-up both within healthy controls and across all cases, including controls as well as SMC, MCI, and AD dementia cases. The lack of effect for the 2-compartmental PVC method is consistent with the notion that explicit modeling of white matter signal spill-in effects is required in order to observe PVC-related sensitivity increases in amyloid-PET data. Likewise, a PVC approach designed to correct for spill-in of gray matter signal into (low-intensity) white matter (Coello et al., 2013) showed no effect on the association of 18F-Flutemetamol-PET signal with CSF Aβ42 levels at low levels of amyloid (Kalheim et al., 2018).

Interestingly, using a white matter reference also resulted in a significantly higher association between 18F-Florbetapir-PET signal and CSF Aβ42 levels compared to the standard processing approach, irrespective of the PVC method. The effect of using a white matter reference was similar to that of 3-compartmental PVC, suggesting that correction for unspecific white matter binding at least partly contributes to the higher associations when using a white matter reference region. Our findings agree with an 18F-Florbetapir-PET study from the ADNI cohort showing less variability in longitudinal SUVR values when computed using a white matter rather than a cerebellar reference region, resulting in a higher sensitivity to detect increase of amyloid uptake over two years (Chen et al., 2015). The use of cerebral white matter as a reference region, however, has major difficulties as well. An 11C-PIB-PET study found a significant association between white matter signal and age. The age effect was more pronounced for subcortical than for periventricular white matter. In addition, in some white matter regions the age effect was stronger for Aβ-positive than for Aβ-negative cases, whereas in other white matter regions it was stronger for Aβ-negative than for Aβ-positive cases (Lowe et al., 2018). The mechanisms underlying the age effect are currently unclear. In a simulation study of 18F-Florbetapir kinetics, white matter signal was more susceptible to variation in cerebral blood flow and fluctuations of the input function than whole cerebellar signal (Kameyama et al., 2019). Following these data, the advantage of white matter signal as a reference region found in some previous studies may come at the cost of higher bias from cerebral blood flow variations, which may also explain part of the effects of age and disease stage on white matter signal. These potential confounds are strong arguments against an uncritical use of white matter signal as a reference.

First regions to show increased 18F-Florbetapir-PET signal at decreasing CSF Aβ42 levels included the inferior temporal gyrus, temporo-occipital areas, precuneus, superior parietal lobe, and dorsal medial prefrontal areas. This pattern was consistent across the different PVC methods. This pattern differs partly from the pattern described in a previous study using ^11^C-PIB-PET data (Villeneuve et al., 2015) where the earliest regions were located in the medial prefrontal cortex and precuneus, while superior parietal lobule and lateral temporal cortex were only involved at higher levels of amyloid. The regions identified in this previous study and ours are entirely included in the brain regions encompassed by Thal phase 1, representing the brain areas with earliest Aβ build-up in autopsy samples (Thal et al., 2018). The regional distribution of Aβ plaques in Thal phase 1, however, is very extensive, including large parts of prefrontal cortex, parietal and lateral temporal lobes, which renders this overlap rather unspecific. Still, the sparing of subcortical areas from early amyloid accumulation in our and the previous study using a similar sliding window approach (Villeneuve et al., 2015) is consistent with the later appearance of amyloid in these structures starting in Thal phase 3 (Thal et al., 2002). The early amyloid accumulating regions in our study also overlap with the regions involved in the earliest stages of amyloid accumulation as determined by previous regional amyloid-PET staging studies. Specifically, our regions overlap with stages 1 and 2 of the four Grothe stages (Grothe et al., 2017), and early and intermediate stages from three Mattsson stages (Mattsson et al., 2019), both based on 18F-Florbetapir-PET. They also overlap with the regions showing the highest frequency of amyloid build-up in 18F-florbetaben-PET data (Cho et al., 2016). As with the early Thal phases, the early amyloid stages in these previous staging studies were spatially much more extended than the earliest regions from our present study, independently from the staging method. The early involvement of the inferior temporal cortex in our study agrees with a previous study showing a significant increase of neocortical temporal lobe signal in 18F-Florbetapir-PET scans of cognitively healthy adults between 20 and 60 years of age (Gonneaud et al., 2017). A recent review (Fantoni et al., 2020) showed that the predominance of involved brain regions differed across studies, with some studies showing a more prefrontal and some a more posterior pattern in the earliest stages. These differences may partly reflect characteristics of different tracers, but regional variations across studies using the same tracer also suggest an important role of the quantification approach.

One may speculate that the sliding window approach employed in this study may be particularly sensitive to uncover the very first regions to show amyloid build-up in early stage amyloidosis, at least as measured by decreasing CSF Aβ42 levels as the reference standard. However, longitudinal data would be required to substantiate such a claim. The number of longitudinal PET studies on the regional spread of amyloid in the early stages of amyloidosis is still limited. The regions detected in our study form a subset of the regions that showed early accumulation of amyloid over two years of follow-up in 18F-Florbetapir-PET data from the ADNI and the BioFINDER cohorts (Palmqvist et al., 2017).

### Limitations

Our study had several limitations. First, the Elecsys Aβ42 assay presents a technical limitation for values above 1,700 pg/ml that renders these higher values exploratory. Therefore, analyses were repeated using the CSF Aβ42 Innogenetics ELISA assay, and these analyses could confirm the PVC-3-related increase in the correspondence between 18F-Florbetapir-PET signal and CSF Aβ42 levels. In addition, we could replicate our key findings in an independent replication sample. Secondly, we would have liked to use the CSF Aβ42/Aβ40 ratio as a reference for the amyloid-PET signal (Pannee et al., 2016). However, CSF Aβ40 values were only available in a small subset of the ADNI data and not in the INSIGHT-preAD study. Thus, this analysis needs to await the availability of larger numbers. Third, the pathogenic interpretation of our findings is difficult. CSF Aβ42 and amyloid sensitive PET using 18F-Florbetapir capture different pools of amyloid. Hence, a stronger correlation between both signals, CSF and PET, does not mean that the PVC-3 corrected amyloid-PET signal captures an amyloid fraction that is closer to the amyloid fraction represented by CSF Aβ42 than the uncorrected PET signal. However, one would expect that the different amyloid fractions are in equilibrium so that capturing more closely one fraction may also increase the accuracy of capturing the fraction which is upstream of it. Fourth, the current findings cannot directly be generalized to other amyloid-PET tracers. One would expect similar effects, since unspecific white matter binding plays a role for all currently approved tracers; however, this remains to be directly verified. Finally, the notion of the earliest involved brain regions should not be interpreted as direct evidence for a longitudinal spread of amyloid, although they appear to be consistent with evidence of regional amyloid spread from recent longitudinal studies (Palmqvist et al., 2017; Jelistratova et al., 2020).

## Conclusion

In summary, both PVC-3 and a white matter reference region markedly increased the match between CSF Aβ42 levels and 18F-Florbetapir-PET signal in the earliest stages of amyloid accumulation. Additionally, we found a regional 18F-Florbetapir-PET pattern of early amyloid build-up in inferior temporal, precuneus and prefrontal cortical regions that was more sensitively detected when accounting for unspecific white matter signal confounds using 3-comparmental PVC. Comparison with longitudinal PET studies suggest that these regions represent a subset of previously defined areas that showed early signs of amyloid accumulation (Palmqvist et al., 2017), but more specific analysis would be required to confirm the early involvement of these regions. As a note of caution, however, the striking increase of sensitivity using a white matter reference may be counterbalanced by higher effects of confounding factors, such as age and diagnosis, on the white matter signal. Thus, in a more conservative approach, one would recommend the use of 3-compartmental PVC with a well-established cerebellar reference region to increase sensitivity of PET scans for early amyloid uptake. Whether such uptake is indeed relevant for mid- to long-term clinical prognosis of individual cases needs to be shown in subsequent longitudinal studies.

## Data Availability Statement

Data used for the current analysis from the ADNI cohort are freely available from the ADNI website (http://adni.loni.usc.edu/data-samples/access-data), including CSF Aβ levels and amyloid PET scans. Processed partial volume corrected PET scans are available upon request through the corresponding authors of this manuscript. Data from the INSIGHT-preAD cohort are only available upon written request and after approval by the INSIGHT-preAD scientific review board.

## Ethics Statement

The studies involving human participants were reviewed and approved by the institutional review boards of the participating institutions. All procedures performed in both ADNI and INSIGHT-preAD studies were in agreement with the ethical standards of the Institutional Research Committees and the 1975 Helsinki Declaration and its later amendments. Written informed consent was obtained from all participants and/or authorized representatives and the study partners before any protocol-specific procedures were carried out in the ADNI or INSIGHT-preAD studies.

## Author Contributions

SJT and MJG have made substantial contributions to the conception and design of the work, the interpretation of the data, and have drafted the work. MD and HH have made substantial contributions to the design of the work, the interpretation of data, and have substantively revised the draft. BD, SL, MOH, and M-CP have made substantial contributions to the conception and design of the work, the acquisition of the data, and have substantively revised the draft. AV and FL have made substantial contributions to the interpretation of the data and have substantively revised the draft. All authors have approved the submitted version and have agreed both to be personally accountable for the author’s own contributions and to ensure that questions related to the accuracy or integrity of any part of the work, even ones in which the author was not personally involved, are appropriately investigated, resolved, and the resolution documented in the literature.

## Conflict of Interest

SJT participated in scientific advisory boards of Roche Pharma AG, Biogen, and MSD, and received lecture fees from Roche and MSD. AV was an employee of Eisai Inc. [November 2019 – Jun 2021]. This work has been performed during his previous position at Sorbonne University, Paris, France. Before November 2019 he had received lecture honoraria from Roche, MagQu LLC, and Servier. SL received lecture honoraria from Roche and Servier. BD received consultant fees from Lilly, Boehringer Ingelheim and has received grants from Roche for his institution. HH is an employee of Eisai Inc. This work has been performed during his previous position at Sorbonne University, Paris, France. He serves as Senior Associate Editor for the Journal Alzheimer’s & Dementia and does not receive any fees or honoraria since May 2019. Before May 2019 he was supported by the AXA Research Fund, the “Fondation partenariale Sorbonne Université” and the “Fondation pour la Recherche sur Alzheimer,” Paris, France. He had received lecture fees from Servier, Biogen and Roche, research grants from Pfizer, Avid, and MSD Avenir (paid to the institution), travel funding from Functional Neuromodulation, Axovant, Eli Lilly and company, Takeda and Zinfandel, GE-Healthcare and Oryzon Genomics, consultancy fees from Qynapse, Jung Diagnostics, Cytox Ltd., Axovant, Anavex, Takeda and Zinfandel, GE Healthcare and Oryzon Genomics, and Functional Neuromodulation, and participated in scientific advisory boards of Functional Neuromodulation, Axovant, Eisai, Eli Lilly and company, Cytox Ltd., GE Healthcare, Takeda and Zinfandel, Oryzon Genomics and Roche Diagnostics. He is co-inventor in the following patents as a scientific expert and has received no royalties: (1) *In vitro* Multiparameter Determination Method for The Diagnosis and Early Diagnosis of Neurodegenerative Disorders Patent Number: 8916388; (2) *In vitro* Procedure for Diagnosis and Early Diagnosis of Neurodegenerative Diseases Patent Number: 8298784; (3) Neurodegenerative Markers for Psychiatric Conditions Publication Number: 20120196300; (4) *In vitro* Multiparameter Determination Method for The Diagnosis and Early Diagnosis of Neurodegenerative Disorders Publication Number: 20100062463; (5) *In vitro* Method for The Diagnosis and Early Diagnosis of Neurodegenerative Disorders Publication Number: 20100035286; (6) *In vitro* Procedure for Diagnosis and Early Diagnosis of Neurodegenerative Diseases Publication Number: 20090263822; (7) *In vitro* Method for The Diagnosis of Neurodegenerative Diseases Patent Number: 7547553; (8) CSF Diagnostic *in vitro* Method for Diagnosis of Dementias and Neuroinflammatory Diseases Publication Number: 20080206797; (9) *In vitro* Method for The Diagnosis of Neurodegenerative Diseases Publication Number: 20080199966; (10) Neurodegenerative Markers for Psychiatric Conditions Publication Number: 20080131921. MOH received honoraria as a consultant from Blue Earth company. The remaining authors declare that the research was conducted in the absence of any commercial or financial relationships that could be construed as a potential conflict of interest. The reviewer GS declared a past co-authorship with one of the authors, MJG, to the handling editor.

## Publisher’s Note

All claims expressed in this article are solely those of the authors and do not necessarily represent those of their affiliated organizations, or those of the publisher, the editors and the reviewers. Any product that may be evaluated in this article, or claim that may be made by its manufacturer, is not guaranteed or endorsed by the publisher.

## Footnotes

1
http://adni.loni.usc.edu/

2
http://adni.loni.usc.edu/methods/

## Abbreviations

AD: Alzheimer’s disease
ADNI: Alzheimer’s Disease Neuroimaging Initiative
CSF: cerebrospinal fluid
FEW: family-wise error
INSIGHT-preAD: INveStIGation of AlzHeimer’s PredicTors cohort
MCI: mild cognitive impairment
MMSE: Mini Mental State Examination
MRI: magnetic resonance imaging
PET: positron emission tomography
PVC: partial volume effect correction
PVE: partial volume effect
SPM: Statistical Parametric Mapping
SUVR: standard uptake value ratio.

## References

[B1] Blennow K. Mattsson N. Scholl M. Hansson O. Zetterberg H. (2015). Amyloid biomarkers in Alzheimer’s disease. *Trends Pharmacol. Sci.* 36 297–309. 25840462 10.1016/j.tips.2015.03.002

[B2] Catafau A. M. Bullich S. Seibyl J. P. Barthel H. Ghetti B. Leverenz J. (2016). Cerebellar amyloid-beta plaques: how frequent are they, and do they influence 18F-florbetaben SUV ratios? *J. Nucl. Med.* 57 1740–1745. 10.2967/jnumed.115.171652 27363836

[B3] Chen K. Roontiva A. Thiyyagura P. Lee W. Liu X. Ayutyanont N. (2015). Improved power for characterizing longitudinal amyloid-beta PET changes and evaluating amyloid-modifying treatments with a cerebral white matter reference region. *J. Nucl. Med.* 56 560–566. 10.2967/jnumed.114.149732 25745091

[B4] Cho H. Choi J. Y. Hwang M. S. Kim Y. J. Lee H. M. Lee H. S. (2016). In vivo cortical spreading pattern of tau and amyloid in the Alzheimer disease spectrum. *Ann. Neurol.* 80 247–258. 10.1002/ana.24711 27323247

[B5] Coello C. Willoch F. Selnes P. Gjerstad L. Fladby T. Skretting A. (2013). Correction of partial volume effect in (18)F-FDG PET brain studies using coregistered MR volumes: voxel based analysis of tracer uptake in the white matter. *Neuroimage* 72 183–192. 10.1016/j.neuroimage.2013.01.043 23370062

[B6] Desikan R. S. Segonne F. Fischl B. Quinn B. T. Dickerson B. C. Blacker D. (2006). An automated labeling system for subdividing the human cerebral cortex on MRI scans into gyral based regions of interest. *Neuroimage* 31 968–980. 10.1016/j.neuroimage.2006.01.021 16530430

[B7] Dubois B. Epelbaum S. Nyasse F. Bakardjian H. Gagliardi G. Uspenskaya O. (2018). Cognitive and neuroimaging features and brain beta-amyloidosis in individuals at risk of Alzheimer’s disease (INSIGHT-preAD): a longitudinal observational study. *Lancet Neurol.* 17 335–346. 10.1016/S1474-4422(18)30029-2 29500152

[B8] Erlandsson K. Buvat I. Pretorius P. H. Thomas B. A. Hutton B. F. (2012). A review of partial volume correction techniques for emission tomography and their applications in neurology, cardiology and oncology. *Phys. Med. Biol.* 57 R119–R159. 10.1088/0031-9155/57/21/R119 23073343

[B9] Fantoni E. Collij L. Alves I. L. Buckley C. Farrar G. Consortium A. (2020). The spatial-temporal ordering of amyloid pathology and opportunities for PET imaging. *J. Nucl. Med.* 61 166–171. 10.2967/jnumed.119.235879 31836683

[B10] Folstein M. F. Folstein S. E. Mchugh P. R. (1975). Mini-mental-state: a practical method for grading the cognitive state of patients for the clinician. *J. Psychiatr. Res.* 12 189–198. 1202204 10.1016/0022-3956(75)90026-6

[B11] Gonneaud J. Arenaza-Urquijo E. M. Mezenge F. Landeau B. Gaubert M. Bejanin A. (2017). Increased florbetapir binding in the temporal neocortex from age 20 to 60 years. *Neurology* 89 2438–2446. 10.1212/WNL.0000000000004733 29150540

[B12] Gonzalez-Escamilla G. Lange C. Teipel S. Buchert R. Grothe M. J. Alzheimer’s Disease (2017). PETPVE12: an SPM toolbox for Partial Volume Effects correction in brain PET - Application to amyloid imaging with AV45-PET. *Neuroimage* 147 669–677. 10.1016/j.neuroimage.2016.12.077 28039094

[B13] Grothe M. J. Barthel H. Sepulcre J. Dyrba M. Sabri O. Teipel S. J. (2017). In vivo staging of regional amyloid deposition. *Neurology* 89 2031–2038. 10.1212/wnl.0000000000004643 29046362 PMC5711511

[B14] Habert M. O. Bertin H. Labit M. Diallo M. Marie S. Martineau K. (2017). Evaluation of amyloid status in a cohort of elderly individuals with memory complaints: validation of the method of quantification and determination of positivity thresholds. *Ann. Nucl. Med.* 32 75–86. 10.1007/s12149-017-1221-0 29218458

[B15] Hoffman E. J. Huang S. C. Phelps M. E. (1979). Quantitation in positron emission computed tomography: 1. Effect of object size. *J. Comput. Assist. Tomogr.* 3 299–308. 10.1097/00004728-197906000-00001 438372

[B16] Jack C. R. Jr. Bennett D. A. Blennow K. Carrillo M. C. Dunn B. Haeberlein S. B. (2018). NIA-AA research framework: toward a biological definition of Alzheimer’s disease. *Alzheimers Dement.* 14 535–562. 10.1016/j.jalz.2018.02.018 29653606 PMC5958625

[B17] Janelidze S. Zetterberg H. Mattsson N. Palmqvist S. Vanderstichele H. Lindberg O. (2016). CSF Abeta42/Abeta40 and Abeta42/Abeta38 ratios: better diagnostic markers of Alzheimer disease. *Ann. Clin. Transl. Neurol.* 3 154–165. 27042676 10.1002/acn3.274 PMC4774260

[B18] Jelistratova I. Teipel S. J. Grothe M. J. (2020). Longitudinal validity of PET-based staging of regional amyloid deposition. *Hum. Brain Mapp.* 41, 4219–4231. 10.1002/hbm.25121 32648624 PMC7502828

[B19] Kalheim L. F. Fladby T. Coello C. Bjornerud A. Selnes P. (2018). [18F]-Flutemetamol uptake in cortex and white matter: comparison with cerebrospinal fluid biomarkers and [18F]-fludeoxyglucose. *J. Alzheimers. Dis.* 62 1595–1607. 10.3233/JAD-170582 29504529 PMC6218124

[B20] Kameyama M. Ishibash K. Wagatsuma K. Toyohara J. Ishii K. (2019). A pitfall of white matter reference regions used in [(18)F] florbetapir PET: a consideration of kinetics. *Ann. Nucl. Med.* 33 848–854. 10.1007/s12149-019-01397-y 31456012

[B21] Klunk W. E. Koeppe R. A. Price J. C. Benzinger T. L. Devous M. D. Sr. Jagust W. J. (2015). The Centiloid Project: standardizing quantitative amyloid plaque estimation by PET. *Alzheimers Dement.* 11 1.e4–15.e4. 10.1016/j.jalz.2014.07.003 25443857 PMC4300247

[B22] Leuzy A. Chiotis K. Hasselbalch S. G. Rinne J. O. De Mendonca A. Otto M. (2016). Pittsburgh compound B imaging and cerebrospinal fluid amyloid-beta in a multicentre European memory clinic study. *Brain* 139 2540–2553. 10.1093/brain/aww160 27401520 PMC4995359

[B23] Lopez-Gonzalez F. J. Moscoso A. Efthimiou N. Fernandez-Ferreiro A. Pineiro-Fiel M. Archibald S. J. (2019). Spill-in counts in the quantification of (18)F-florbetapir on Abeta-negative subjects: the effect of including white matter in the reference region. *EJNMMI Phys.* 6:27. 10.1186/s40658-019-0258-7 31858289 PMC6923310

[B24] Lowe V. J. Lundt E. S. Senjem M. L. Schwarz C. G. Min H. K. Przybelski S. A. (2018). White matter reference region in PET studies of (11)C-pittsburgh compound B uptake: effects of age and amyloid-beta deposition. *J. Nucl. Med.* 59 1583–1589. 10.2967/jnumed.117.204271 29674420 PMC6167534

[B25] Martinez G. Vernooij R. W. Fuentes Padilla P. Zamora J. Bonfill Cosp X. Flicker L. (2017). 18F PET with florbetapir for the early diagnosis of Alzheimer’s disease dementia and other dementias in people with mild cognitive impairment (MCI). *Cochrane Database Syst. Rev.* 11:CD012216. 10.1002/14651858.CD012216.pub2 29164603 PMC6486090

[B26] Matsubara K. Ibaraki M. Shimada H. Ikoma Y. Suhara T. Kinoshita T. (2016). Impact of spillover from white matter by partial volume effect on quantification of amyloid deposition with [(11)C]PiB PET. *Neuroimage* 143 316–324. 10.1016/j.neuroimage.2016.09.028 27639351

[B27] Mattsson N. Palmqvist S. Stomrud E. Vogel J. Hansson O. (2019). Staging beta-amyloid pathology with amyloid positron emission tomography. *JAMA Neurol.* 76 1319–1329. 10.1001/jamaneurol.2019.2214 31314895 PMC6646987

[B28] Meltzer C. C. Zubieta J. K. Links J. M. Brakeman P. Stumpf M. J. Frost J. J. (1996). MR-based correction of brain PET measurements for heterogeneous gray matter radioactivity distribution. *J. Cereb. Blood Flow Metab.* 16 650–658. 10.1097/00004647-199607000-00016 8964805

[B29] Mikhno A. Devanand D. Pelton G. Cuasay K. Gunn R. Upton N. (2008). Voxel-based analysis of 11C-PIB scans for diagnosing Alzheimer’s disease. *J. Nucl. Med.* 49 1262–1269. 18632806 10.2967/jnumed.107.049932 PMC3103049

[B30] Müller-Gärtner H. W. Links J. M. Prince J. L. Bryan R. N. Mcveigh E. Leal J. P. (1992). Measurement of radiotracer concentration in brain gray matter using positron emission tomography: MRI-based correction for partial volume effects. *J. Cerbr. Blood Flow Metab.* 12 571–583. 10.1038/jcbfm.1992.81 1618936

[B31] Navitsky M. Joshi A. D. Kennedy I. Klunk W. E. Rowe C. C. Wong D. F. (2018). Standardization of amyloid quantitation with florbetapir standardized uptake value ratios to the Centiloid scale. *Alzheimers Dement.* 14 1565–1571. 10.1016/j.jalz.2018.06.1353 30006100

[B32] Palmqvist S. Mattsson N. Hansson O. Alzheimer’s Disease Neuroimaging Initiative (2016). Cerebrospinal fluid analysis detects cerebral amyloid-beta accumulation earlier than positron emission tomography. *Brain* 139 1226–1236. 10.1093/brain/aww015 26936941 PMC4806222

[B33] Palmqvist S. Scholl M. Strandberg O. Mattsson N. Stomrud E. Zetterberg H. (2017). Earliest accumulation of beta-amyloid occurs within the default-mode network and concurrently affects brain connectivity. *Nat. Commun.* 8:1214. 10.1038/s41467-017-01150-x 29089479 PMC5663717

[B34] Pannee J. Portelius E. Minthon L. Gobom J. Andreasson U. Zetterberg H. (2016). Reference measurement procedure for CSF amyloid beta (Abeta)1-42 and the CSF Abeta1-42/Abeta1-40 ratio - a cross-validation study against amyloid PET. *J. Neurochem.* 139 651–658. 10.1111/jnc.13838 27579672

[B35] Rullmann M. Dukart J. Hoffmann K. T. Luthardt J. Tiepolt S. Patt M. (2016). Partial-volume effect correction improves quantitative analysis of 18F-florbetaben beta-amyloid PET scans. *J. Nucl. Med.* 57 198–203. 10.2967/jnumed.115.161893 26541776

[B36] Schmidt M. E. Chiao P. Klein G. Matthews D. Thurfjell L. Cole P. E. (2015). The influence of biological and technical factors on quantitative analysis of amyloid PET: points to consider and recommendations for controlling variability in longitudinal data. *Alzheimers Dement.* 11 1050–1068. 10.1016/j.jalz.2014.09.004 25457431

[B37] Shaw L. M. Vanderstichele H. Knapik-Czajka M. Clark C. M. Aisen P. S. Petersen R. C. (2009). Cerebrospinal fluid biomarker signature in Alzheimer’s disease neuroimaging initiative subjects. *Ann. Neurol.* 65 403–413. 10.1002/ana.21610 19296504 PMC2696350

[B38] Spallazzi M. Barocco F. Michelini G. Immovilli P. Taga A. Morelli N. (2019). CSF biomarkers and amyloid PET: concordance and diagnostic accuracy in a MCI cohort. *Acta Neurol. Belg.* 119 445–452. 10.1007/s13760-019-01112-8 30847669

[B39] Teipel S. Heinsen H. Amaro E. Jr. Grinberg L. T. Krause B. Grothe M. (2014). Cholinergic basal forebrain atrophy predicts amyloid burden in Alzheimer’s disease. *Neurobiol. Aging* 35 482–491. 10.1016/j.neurobiolaging.2013.09.029 24176625 PMC4120959

[B40] Thal D. R. Beach T. G. Zanette M. Lilja J. Heurling K. Chakrabarty A. (2018). Estimation of amyloid distribution by [(18)F]flutemetamol PET predicts the neuropathological phase of amyloid beta-protein deposition. *Acta Neuropathol.* 136 557–567. 10.1007/s00401-018-1897-9 30123935 PMC6132944

[B41] Thal D. R. Rub U. Orantes M. Braak H. (2002). Phases of A beta-deposition in the human brain and its relevance for the development of AD. *Neurology* 58 1791–1800. 10.1212/wnl.58.12.1791 12084879

[B42] Toledo J. B. Bjerke M. Da X. Landau S. M. Foster N. L. Jagust W. (2015). Nonlinear association between cerebrospinal fluid and florbetapir f-18 beta-amyloid measures across the spectrum of Alzheimer disease. *JAMA Neurol.* 72 571–581. 10.1001/jamaneurol.2014.4829 25822737 PMC5642905

[B43] Villeneuve S. Rabinovici G. D. Cohn-Sheehy B. I. Madison C. Ayakta N. Ghosh P. M. (2015). Existing Pittsburgh Compound-B positron emission tomography thresholds are too high: statistical and pathological evaluation. *Brain* 138 2020–2033. 10.1093/brain/awv112 25953778 PMC4806716

[B44] Zhang S. Smailagic N. Hyde C. Noel-Storr A. H. Takwoingi Y. Mcshane R. (2014). (11)C-PIB-PET for the early diagnosis of Alzheimer’s disease dementia and other dementias in people with mild cognitive impairment (MCI). *Cochrane Database. Syst. Rev.* 7:CD010386. 10.1002/14651858.CD010386.pub2 25052054 PMC6464750

