# Supplementary material for: Partial Volume Correction Increases the Sensitivity of 18F-Florbetapir-Positron Emission Tomography for the Detection of Early Stage Amyloidosis
Source: Front Aging Neurosci. 2021 Dec 22;13:748198. doi: 10.3389/fnagi.2021.748198 (PMC8729321; doi:10.3389/fnagi.2021.748198)
Supplement: Supplementary file 1 [file Data_Sheet_1.docx]

**Supplementary material section**

**Sensitivity analyses**

Since the upper technical limit of the Elecsys assay for CSF Aβ42 is 1700 pg/ml and values above this threshold arise from an extrapolation of the calibration curve*,* we used previously established CSF Aβ42 values from an Innogenetics ELISA assay for sensitivity analysis.

Replicating the initial findings derived from the Elecsys assay, PVC-3 again showed significantly higher negative spline estimates and significantly more negative correlations between CSF Aβ42 levels and low range 18F-Florbetapir-PET SUVR_WC_ values (between the 16.6% quantile and first tertile) compared to no PVC and PVC-2 (Supplementary Figures 1 and 2). Moreover, for the white matter reference the associations were again more negative than for the whole cerebellum standard processing, and associations were comparable across PVC methods in this data.

For sensitivity analysis of the voxel-wise sliding window approach, we defined the reference group with highest CSF Aβ42 levels using the upper 10% or 20% quantiles instead of the initially chosen 15% quantile. As expected, the corresponding CSF thresholds at which first regional signal elevations became statistically significant varied with the size of the reference sample (which also determined the size of the sliding group of interest). Thus, when defining the reference sample using the 10% quantile (n = 15 cases) first statistically significant 18F-Florbetapir-PET signal elevations in the sliding group of interest were observed at lower mean CSF Aβ42 levels when compared to using the 15% quantile (n = 23 cases) as the reference sample, whereas these mean CSF Aβ42 levels were higher when using the 20% quantile as reference sample (n = 30 cases). However, for any given reference sample, mean CSF Aβ42 levels at which first significant 18F-Florbetapir-PET signal elevations appeared were always higher (less pathologic) for PVC-3 compared to no PVC or PVC-2 (supplementary Table 1). Moreover, the regional distribution of the first signal elevations was consistent across the different reference samples based on the upper 10%, 15% or 20% quantiles (data not shown).

**Supplementary Table 1: Mean CSF Aβ42 levels of early 18Florebetapir-PET signal with different PVC methods and sample sizes**

| **Quantiles of reference sample** | **Sample size of reference/index samples** | **PVC method** | **Mean CSF Aβ42 level for reference sample (pg/ml)** | **Mean CSF Aβ42 level for index sample (pg/ml)** |
| --- | --- | --- | --- | --- |
| 10^th^ | N = 15 | No PVC | 2,663 | 817 |
|  |  | PVC-2 |  | 775 |
|  |  | PVC-3 |  | 865 |
| 15^th^ | N = 23 | No PVC | 2,512 | 840 |
|  |  | PVC-2 |  | 840 |
|  |  | PVC-3 |  | 928 |
| 20^th^ | N = 30 | No PVC | 2,494 | 881 |
|  |  | PVC-2 |  | 881 |
|  |  | PVC-3 |  | 993 |

**Supplementary Figure legends:**

**Supplementary Figure 1: Linear spline coefficients for early stage amyloidosis**

Boxplots of linear spline coefficients from regression of the 18F-Florbetapir-PET signal on CSF Aβ42 levels (from Innogenetics assay) in the ADNI sample with the range sliding between the 16% quantile and the first tertile of 18F-Florbetapir-PET values.

WhCer - whole cerebellum reference region

WM - white matter reference region

**Supplementary Figure 2: Correlation coefficients for early stage amyloidosis**

Boxplots of correlation coefficients between 18F-Florbetapir-PET signal and CSF Aβ42 levels (from Innogenetics assay) in the ADNI sample with the range sliding between the 16% quantile and the first tertile of 18F-Florbetapir-PET values.

WhCer - whole cerebellum reference region

WM - white matter reference region

**Supplementary Figure 3: Linear spline estimates for CSF Aβ42 vs. 18F-Florbetapir-PET SUVR in the replication sample**

Spline regression with 95% confidence interval (dashed lines) of the 18F-Florbetapir-PET signal on CSF Aβ42 levels in the replication sample using two knots at the first and the second tertiles of the range of 18F-Florbetapir-PET values. The 18F-Florbetapir-PET and CSF Aβ42 levels were scaled to a mean of 0 and a standard deviation of 1 to render models comparable across PVC methods.

Upper row: w/o (without) PVC,

Lower row: PVC-3.

**Supplementary Figure 1: Linear spline coefficients for early stage amyloidosis**


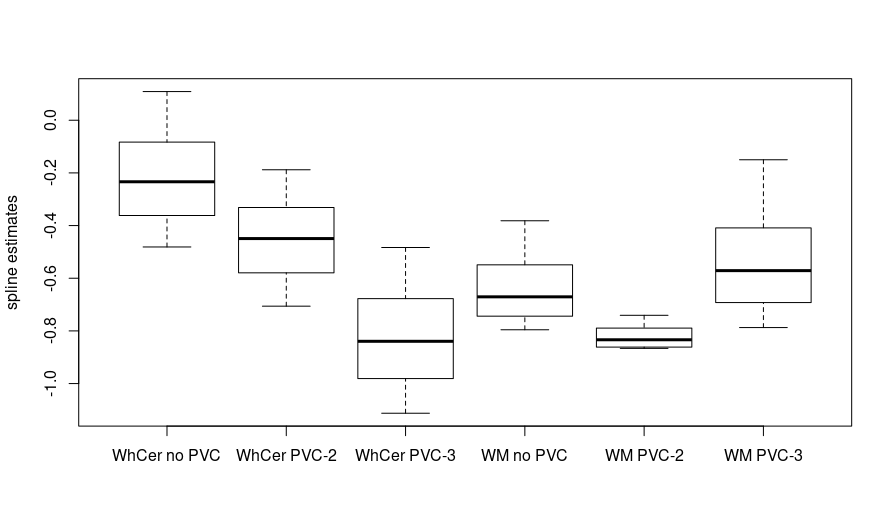


**Supplementary Figure 2: Correlation coefficients for early stage amyloidosis**


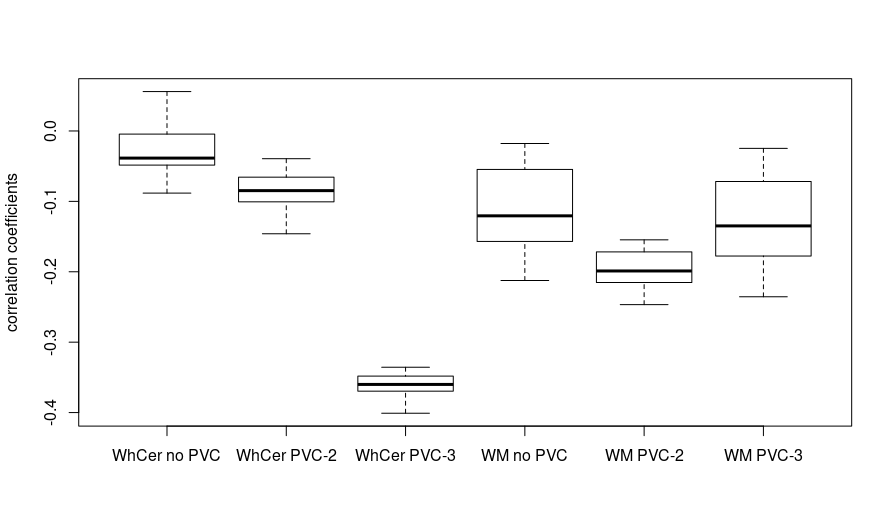


**Supplementary Figure 3: Linear spline estimates for CSF Aβ42 vs. 18F-Florbetapir-PET SUVR in the replication sample**

**
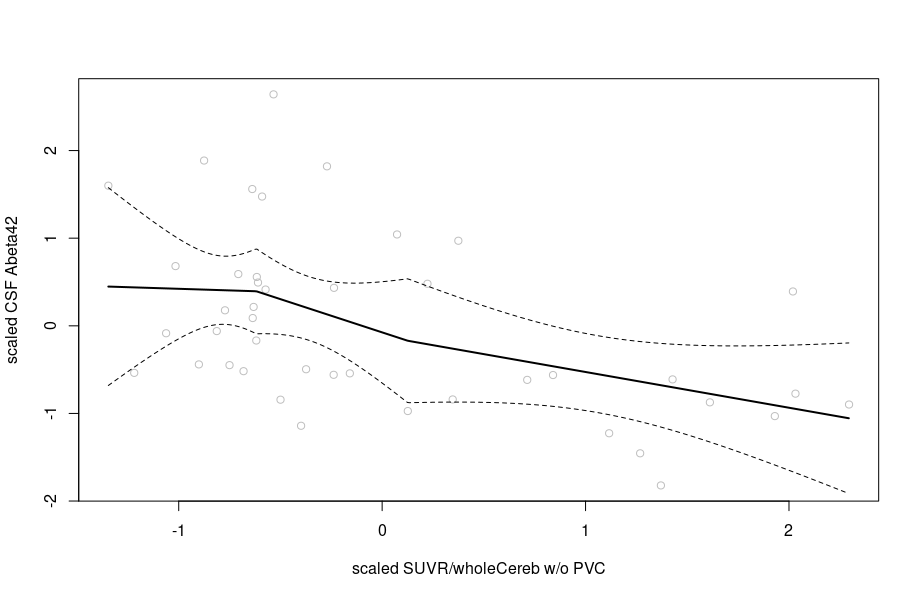
**

**
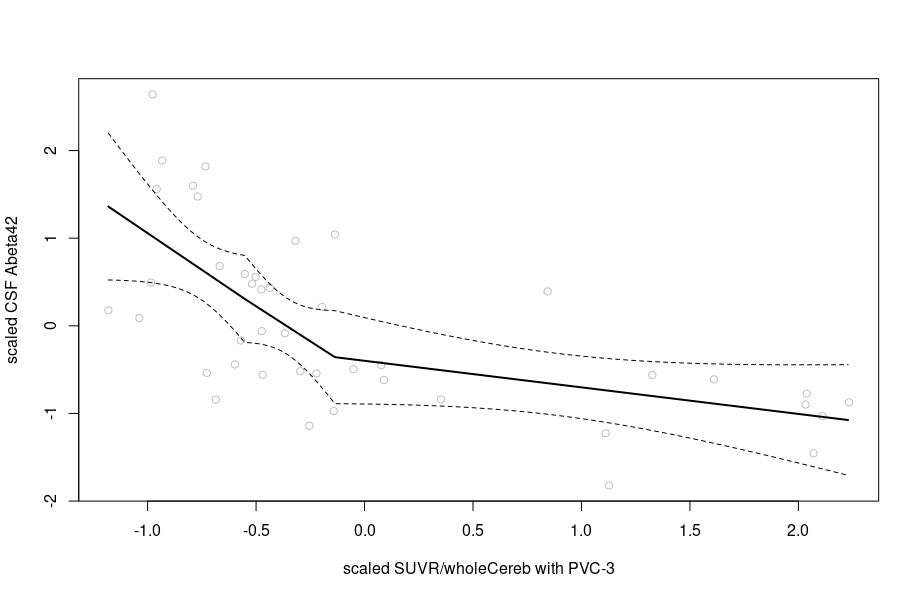
**
